# Effect of intrabronchial administration of autologous adipose-derived mesenchymal stem cells on severe equine asthma

**DOI:** 10.1186/s13287-022-02704-7

**Published:** 2022-01-21

**Authors:** Neža Adamič, Sonja Prpar Mihevc, Rok Blagus, Petra Kramarič, Uroš Krapež, Gregor Majdič, Laurent Viel, Andrew M. Hoffman, Dorothee Bienzle, Modest Vengust

**Affiliations:** 1 Veterinary Faculty, University of Ljubljana, 1000 Ljubljana, Slovenia; 2 Institute for Biostatistics and Medical Informatics, Faculty of Medicine, University of Ljubljana, 1000 Ljubljana, Slovenia; 3 Clinical Studies, University of Guelph, Guelph, ON Canada; 4 School of Veterinary Medicine, University of Pennsylvania, Philadelphia, PA USA; 5 Department of Pathobiology, University of Guelph, Guelph, ON Canada

**Keywords:** Severe equine asthma, Heaves, Pulmonary inflammation, Immune response, Interleukins, Bronchoalveolar lavage fluid, Regenerative medicine, Mesenchymal stem cells

## Abstract

**Background:**

Severe equine asthma (SEA) is a common chronic respiratory disease and a significant health and well-being problem in horses. Current therapeutic strategies improve pulmonary function and clinical signs in some horses, but in the long-term, return to full athletic function appears to be rare. The aim of this study was to assess the safety and the effect of intrabronchial administration of adipose-derived mesenchymal stem cells (AD-MSC) on pulmonary inflammatory and clinical parameters in horses with SEA.

**Methods:**

This was a randomized controlled trial. Twenty adult horses diagnosed with SEA were randomly divided into two groups (*n* = 10), and treated either with a single intrabronchial application of autologous AD-MSC or oral dexamethasone for three weeks. A targeted clinical examination with determination of clinical score, maximal change in pleural pressure during the breathing cycle, and an endoscopic examination of the airways were performed at baseline and three weeks after treatment. Bronchoalveolar lavage fluid was analyzed cytologically, and IL-1β, IL-4, IL-8, IL-17, TNFα and IFNγ mRNA and protein concentrations were measured at baseline and three weeks. The horses were then monitored over one year for recurrence of SEA. A non-inferiority analysis and a linear mixed-effects model were performed to assess differences between treatments.

**Results:**

The non-inferiority of AD-MSC treatment was not established. However, AD-MSC administration significantly ameliorated the clinical score (*P* = 0.01), decreased the expression of IL-17 mRNA (*P* = 0.05) and IL-1β (*P* ≤ 0.001), IL-4 (*P* ≤ 0.001), TNFα (*P* = 0.02) protein levels, and had a positive long-term effect on SEA-associated clinical signs (*P* = 0.02).

**Conclusions:**

Intrabronchial administration of AD-MSC had limited short-term anti-inflammatory effects but improved the clinical signs of SEA at one year.

**Supplementary Information:**

The online version contains supplementary material available at 10.1186/s13287-022-02704-7.

## Background

Severe equine asthma (SEA) is a common chronic respiratory disease of recurrent nature that poses a substantial well-being problem in horses [1–5]. Similar asthma-like conditions occur in humans [4, 6] and cats [7, 8]. Cardinal clinical features are attributed to airway hyperreactivity (AHR) and the underlying inflammatory response of the airways, which is characterized by neutrophilic influx, mucus accumulation, and reversible airway obstruction and remodeling [1, 9]. Traditionally, the management of SEA has included the implementation of changes that reduce exposure to airborne inflammatory stimuli in the equine environment and treatment with topical or systemic anti-inflammatory drugs and bronchodilators [1]. Current therapeutic strategies successfully alleviate clinical signs, but do not adequately treat airway wall remodeling [1, 2, 9–11]. Treatment with systemic corticosteroids may also be associated with adverse effects [12–15]. Inhalation treatment with corticosteroids, for which no significant side effects have been reported, has become a widely used and effective treatment for controlling the clinical signs of SEA [1, 2, 9, 16]. Nevertheless, regardless of the treatment protocol, the majority of treated horses continue to experience episodes of SEA, and approximately half of them remain athletically impaired after disease exacerbation [3].

Regenerative cell-based therapy is becoming an increasingly important treatment option for a variety of clinical problems [17, 18]. Mesenchymal stem cells (MSC), which are undifferentiated cells that can self-renew and transform into tissue cells with specialized functions, are present in the adult organism and are easily obtainable [19]. Therapeutic capabilities of MSC are primarily attributed to immunomodulatory properties that influence the recipient's immune system [20]. Numerous recent studies have investigated the influence of MSC on lung tissue and the lung immune system in animals and humans [21–24]. It has been shown that treatment with MSC alters the local immune response in induced or naturally acquired lung diseases in mouse models [25–29], dogs [30], cats [31] and a sheep model [32, 33]. So far, only one study in horses with SEA has attempted regenerative therapy using intratracheal injection of autologous bone marrow-derived mononuclear cells (BMMC) rather than MSC. A significant improvement in respiratory effort and reduction in inflammation manifest with reduced neutrophilia and IL-10 levels was reported in horses treated with BMMC [34].

The traditional management of SEA only temporarily controls disease exacerbations in most, but not all, horses [5, 35]. Based on the data available to date, it was considered plausible that MSC treatment could have a positive effect without significant side effects. Therefore, the aim of this study was to investigate the effects of intrabronchial administration of MSC derived from autologous adipose tissue (AD-MSC) in horses with SEA. We hypothesized that intrapulmonary treatment with autologous AD-MSC would have a beneficial immunomodulatory effect and induce durable improvement of clinical signs in horses with SEA.

## Materials and methods

This was a randomized controlled trial. All participants in this study were blinded from knowing which treatment horses received. A dedicated study administrator was assigned to manage the study protocol (treatments) and was not linked to any other study procedures. The direct study participants were unblinded after all clinical and laboratory experiments had been completed and the data for statistical analysis had been collected. The unblinding of horse owners/trainers took place after the information on the long term effects of the treatment was obtained.

### Animals

Twenty mature horses (median age 15.2, range from 8 to 23 years), representing seven different breeds (four Quarter Horses, two Lipizzaners, two Icelandic horses, two Shetland ponies, one Italian trotter, one Argentine horse, and eight mix-breeds), were included in this study. The study inclusion criteria were signs of asthma exacerbation following antigen challenge, and reversal of airway obstruction with removal of antigen.

### Outline of the experimental protocol

All horses included in the study were clinical patients with a documented history of SEA. The horses were included in the study with the written consent of their owners. All owners were familiarized with the study protocol and possible adverse effects of the treatments before giving their consent.

During their participation in the study, all horses were kept in the same research facility under uniform conditions. To aggravate and maintain SEA, moldy, dusty hay was shaken in front of the horses twice a day. The maximal change in pleural pressure during the breathing cycle (Δ*P*pl_max_) was measured, and after reversible bronchoconstriction was detected, adipose tissue from the rump of each horse was harvested. The diagnosis of SEA was based on the horse's medical history and clinical examination, including the clinical SEA score, absence of hematological alterations, measurements of ΔPpl_max_, endoscopy of the airways and cytologic evaluation of bronchoalveolar lavage fluid (BALf). Cytokine (IL-1β, IL-4, IL-8, IL-17, TNFα, IFNγ) expression and concentrations in BALf samples were also measured. The horses were then randomly divided into two groups (*n* = 10/group) and treated with either MSC (stem cell treatment group: SCT) or dexamethasone (dexamethasone treatment group; DEX). Autologous AD-MSC re-suspended in phosphate buffered saline (PBS, Gibco, USA, cat. no: 14040174) in the SCT group, or placebo (same volume of PBS without AD-MSC) in the DEX group, were injected endoscopically into the lung's right accessory lobe (time 1: T1). Horses with intrabronchial placebo treatment were then treated with dexamethasone mixed into blended grains (Strucomix Original, Cavalor) at an initial dose of 0.165 mg/kg BW PO q24h for three days, then gradually reduced to 0.083 mg/kg BW PO q24h for the following three days, and further to 0.04 mg/kg BW PO q24h for eight days, 0.02 mg/kg BW PO q24h for three days and finally 0.02 mg/kg BW PO q48h for four days [36–38]. The horses in the SCT group received an equal amount of blended grains without dexamethasone. The horses were re-evaluated three weeks after starting treatment (time 2: T2) and variables (clinical score, Δ*P*pl_max_, endoscopic score, BALf cytology findings, and BALf cytokine expression and concentrations) were again determined.

### Clinical scoring

The clinical condition was determined for each animal at T1 and T2, and the information was used to establish a clinical score. Based on a pre-set scale, a clinical score between 0 and 26 was determined for each horse by assessing respiratory rate, nasal discharge, abdominal expiratory lift, nasal flaring, tracheal sounds, bronchial tones, presence of crackles and wheezes, cough reflex, chest resonance, heart rate and rectal temperature. A higher score indicated a more severe clinical disease [39].

### Measurement of pleural pressure changes

The Δ*P*pl_max_ was measured [40–42] using an esophageal balloon catheter. The catheter was inserted through the horse's nose, advanced through the ventral nasal passage and introduced into the middle thoracic part of the esophagus. After the balloon was placed at the predetermined location, it was inflated with 20 mL of air. The pressure differences in the balloon were transmitted to a portable detection system Venti Graph (Boehringer Ingelheim Vetmedica; Ingelheim/Boucke GmbH u. Co., Germany). The average Δ*P*pl_max_ of 10–15 representative breaths was determined. ΔPpl_max_ values of more than five cmH_2_O were considered as airway obstruction. The esophageal balloon catheter length from the nostrils to the tip of the balloon was recorded for each horse to ensure equal catheter position at *T*1 and *T*2.

Once the airway obstruction was confirmed, the horse was treated intravenously with N-butylscopolammonium bromide (Buscopan® compositum, Boehringer Ingelheim, Germany) at a dose of 0.3 mg/kg BW to confirm the reversibility of the obstruction [43].

### Adipose tissue collection

The adipose tissue was surgically removed from the subcutaneous fat on the rump. The collection site was clipped, aseptically prepared, and locally anesthetized with 20 mL of 2% lidocaine (Xylocaine, AstraZeneca, UK). Approximately three to four grams of well-vascularized adipose tissue was excised. The samples were collected in sterile containers with 15 mL of pre-cooled transport Dulbecco – modified eagle medium (DMEM, Gibco, USA, cat. no: 21885108) and immediately transported to the laboratory.

### Isolation and culture of AD-MSC

The adipose tissue was washed with PBS and minced finely with the scalpel. The adipose tissue was digested in 0.1% type II collagenase (Sigma—Aldrich, DE, cat. no: C1764) overnight at 37 °C with shaking. The digested tissue was then centrifuged at 1600 rpm for four minutes. The cell pellets and the undigested adipose tissue were plated in six-well plates (TPP, Switzerland) in a culture medium containing DMEM (Gibco, USA) and 10% fetal bovine serum (FBS, VWR, USA, cat. no: 97068–085). The cells were cultured at 37 °C in saturated humidity and 5% CO_2_. The cell culture medium was changed every two to three days. The cells were cultured up to the second or third passage until they had multiplied to 100 million cells. They were then harvested, washed at least three times with PBS, and counted using an automatic image-based cell counter (CytoSMART cell counter, Corning Incorporated, Netherlands). The suspension containing 100 million cells was centrifuged and the pellet was resuspended in 30 mL of PBS and transferred to a sterile 60 mL cloaked syringe. There are no established protocols for intrabronchial administration of MSC in horses. Therefore, in this study, 100 million autologous AD-MSC, diluted in 30 mL of PBS, were administered through the catheter (inserted through the endoscope's working channel) into the right accessory lobe of the horse lung.

**Fig. 1:**
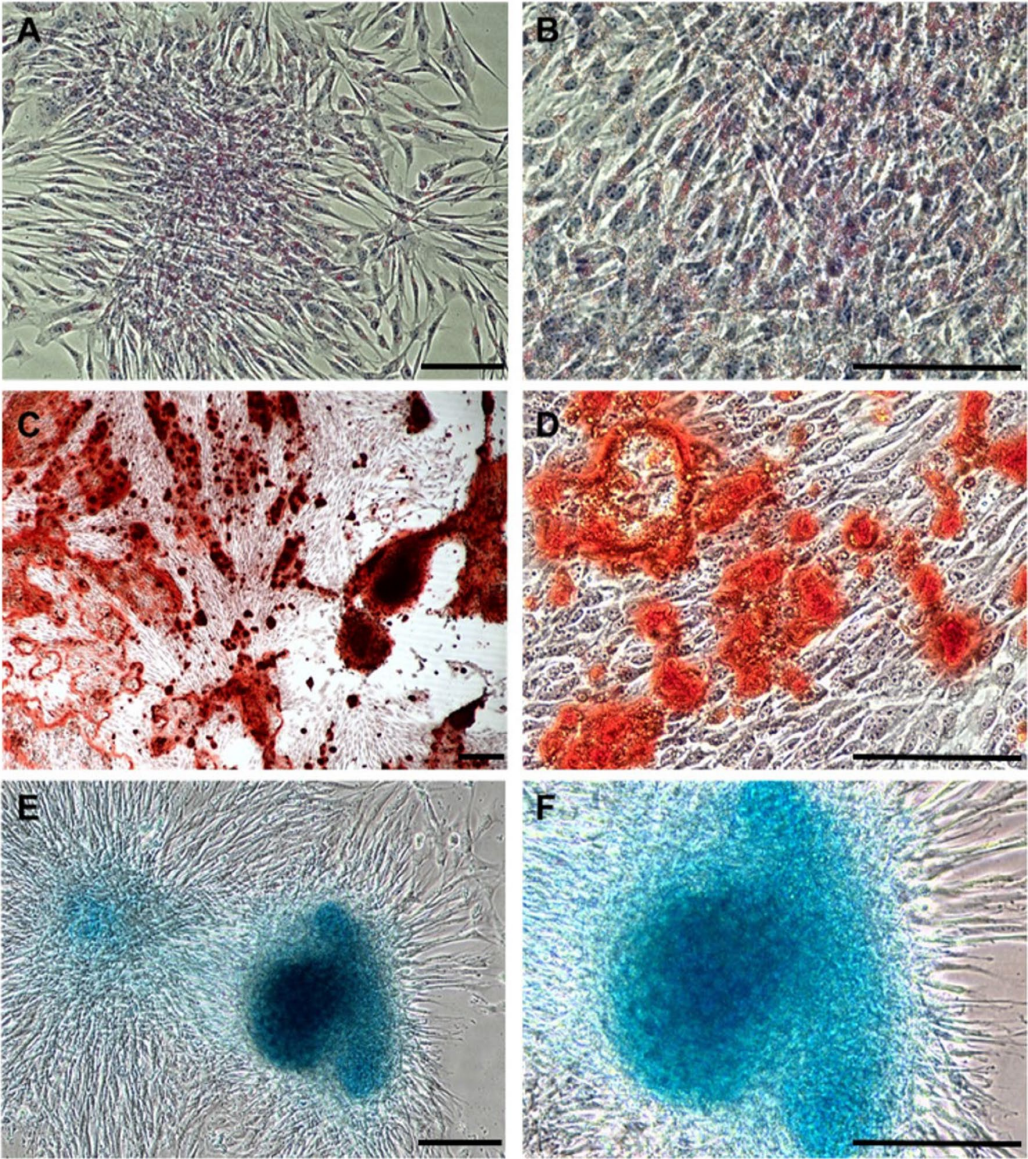
Multilineage differentiation potential of equine adipose derived (AD)-MSCs. Adipogenic differentiation of equine AD-MSC (**a**, **b**); staining of intracytoplasmic lipid droplets with Oil-Red-O and cell nuclei with HE at × 100 (**a**) and × 200 (**b**) magnification (scale bar 100 µm). Osteogenic differentiation of equine AD-MSC (**c**, **d**); staining of calcium matrix formation with Alizarin Red S at × 40 (**c**) and × 200 (**d**) magnification (scale bar 100 µm). Chondrogenic differentiation of equine AD-MSC (**e**, **f**); staining of the cartilage matrix with Alcian Blue at × 100 (**e**) and × 200 (**f**) magnification (scale bar 100 µm)

An aliquot of the cells was maintained in culture to demonstrate the multi-lineage differentiation potential of MSC. Adipogenic, osteogenic and chondrogenic differentiation potential was confirmed by induction of cell differentiation to adipose (StemPro Adipogenesis Differentiation Kit, Gibco, USA, cat. No. A1007001), osteocytic (StemPro Osteogenesis Differentiation Kit, Gibco, USA, cat. No. A1007201), and chondrocytic (StemPro Chondrogenesis Differentiation Kit, Gibco, USA, cat. No. A1007101) cell types (Fig. 1).

### Endoscopic examination, bronchoalveolar lavage fluid collection, and cytology

Prior to endoscopy of the respiratory tract and collection of BALf samples, the horses received a bronchodilator inhalation (five to six puffs, Ventolin, 100 µg/puff, GSK, UK). They were then sedated with 0.01 mg/kg BW of detomidine (Domidine 10 mg/mL, Dechra Veterinary Products, UK) and 0.02 mg/kg BW of butorphanol (Butomidor® 10 mg/mL, Richterpharma, Austria), which were administered intravenously.

An endoscope (Karl Storz – Endoscope, Karl Storz GmbH & Co, Germany) was inserted through the nostrils and passed through the ventral nasal passage to visualize the upper airways, trachea and main bronchi. The upper airways were anesthetized with 30 mL of 0.4% lidocaine solution before passage into the trachea. The endoscope was advanced to the right accessory lobar bronchus, where the bronchoalveolar lavage (BAL) was performed. Sterile warm (37 °C) saline (500 mL) was infused and immediately aspirated through the endoscope's working channel [44]. The amount and macroscopic appearance of the recovered BALf were recorded and the sample was placed in a sterile specimen cup on ice. Based on a predetermined scale, an endoscopic score between 0 and 15 was obtained for each horse by assessing the presence and degree of airway erythema and edema, secretions, hemorrhagic exudate and cough reflex. A higher score indicated more severe clinical disease [39].

The BALf sample was processed within one hour after collection. The total cell count (TCC) was determined with an automatic veterinary hematology analyzer (scil Vet abc Plus+, Horiba, Poland). BALf aliquots of 200 µL were then centrifuged for six minutes at 600 rpm (~ 40 g) in a cytocentrifuge (Centric 250, Domel, Slovenia) and stained with Giemsa stain. A differential cell count of at least 400 consecutive leukocytes was performed at 600× magnification. Epithelial cells and red blood cells were enumerated separately.

The remaining BALf was centrifuged at 1600 rpm for four minutes, and the supernatant and the cell pellets were quickly frozen with liquid nitrogen and stored at − 80 °C for further analysis.

### RNA extraction, reverse transcription and quantitative polymerase chain reaction analysis of IL-1β, IL-4, IL-8, IL-17, TNFα, and IFNγ

The total RNA of BALf cell pellets was extracted with Trizol (TRIzol™Reagent, Thermo Fisher Scientific, USA, cat. no: 15596026) according to the manufacturer's protocol. For each sample, the extracted total RNA concentration and purity were measured with a UV spectrophotometer (NanoDrop™ Lite Spectrophotometer).

A reverse transcription-quantitative polymerase chain reaction was performed on all BALf cell pellet samples. Initially, two µg of total RNA of each sample was reverse transcribed into cDNA using a cDNA reverse transcription kit (High Capacity cDNA Reverse Transcription Kit, Applied Biosystems, Thermo Fisher Scientific, USA, cat. no: 4368814) according to the manufacturer's protocol. All reactions were performed in a total volume of 20 µL. Each sample contained 100 ng/µL of cDNA. The reverse transcription was performed with a thermal cycler (2720 Thermo Cycler, Applied Biosystems, Thermo Fisher Scientific, USA) under the conditions recommended in the manufacturer's protocol (25 °C for 10 min, 37 °C for 120 min, 85 °C for 5 min and finally cooled down to 4 °C) and the samples were stored at -20 °C.

The expression of IL-1β, IL-4, IL-8, IL-17, TNFα, and IFNγ mRNA was measured by quantitative PCR with equine GAPDH used as the reference gene [45]. First, standard curves with serial cDNA dilutions were prepared for each gene of interest and reference gene. The slopes of the regression lines were used to calculate the amplification efficiency for each gene. Relative quantification [46] was carried out using the PCR master mix (TaqMan® Universal Master Mix with UNG, Applied Biosystems, Thermo Fisher Scientific, USA, cat.no: 4440038) and gene expression assays (TaqMan® Gene Expression Assays, Applied Biosystems, Thermo Fisher Scientific, USA, cat.no: 4331182; listed in Additional file 4). The results were analyzed using the Pfaffl method and expressed as the ratio of the gene of interest to the reference gene [47, 48]. The LightCycler 96 (Roche Diagnostics GmbH, Germany) was used for amplification and data collection. The samples were amplified in 96-well plates under the following conditions: 50 °C for 2 min, 95 °C for 10 min, and 40 cycles at 95 °C for 15 s and 60 °C for 60 s. All samples were run in triplicate with individual total volumes of 20 µL using a 20 ng cDNA template. Samples obtained from the same horse before and after therapy were analyzed on the same plate.

### Cytokine quantification by enzyme-linked immunosorbent assay

The ELISA was performed using Nori Equine IL -1β, IL -4, IL -8, IL -17, TNFα and IFNγ ELISA kits for BAL samples (Genorise Scientific, USA, cat.no: GR106041-BAL, GR106025-BAL, GR106476-BAL, GR106717-BAL, GR106004-BAL, and GR106017-BAL, respectively) according to the manufacturer's protocols. Lower–upper detection limits were 94–6000, 31–2000, 10–600, 94–6000, 31–2000 and 15–1000 pg/mL for IL-1β, IL-4, IL-8, IL-17, TNFα and IFNγ, respectively. Intra-assay variations were 6% for IL-1β, IL-4, IL-8, IL-17 and TNFα ELISA kits, and 7% for IFNγ ELISA kit. The inter-assay variations were 9% for IL-4, IL-8, IL-17, TNFα and IFNγ ELISA kits, and 11% for IL-1β ELISA kit. Supernatants of BALf samples were used for ELISA assays. Standards with known concentrations were prepared and each sample was transferred to the wells. All samples were analyzed in duplicates. After incubation at room temperature, the plates were washed several times with Assay Buffer, working dilutions of the detection antibodies were added to each well, and the plate was incubated at room temperature. After incubation and washing, Conjugate Solution and Substrate Solution were added and the reaction was stopped with a Stop Solution. According to the manufacturer's instructions, optical density was measured in a microplate reader (SunriseTM, Tecan Trading AG, Switzerland) set at 450 nm with wavelength correction set at 540 nm. A standard curve was generated by plotting the mean absorbance (optical density) for each standard against the concentration (pg/mL) using software to derive a four-parameter polynomial curve-fit.

### Long-term outcome

The horses were released from the research facility to their original environment after *T*2 and observed by their owners/trainers and local veterinarians.

SEA in the horses that participated in this study was related to their original (home) environment. Therefore, the owners were instructed not to make any changes in their husbandry until the completion of the study, which was monitored throughout by the investigators. The study was terminated if the horses re-developed SEA clinical signs or, for horses that did not show SEA clinical signs, at the predetermined end date of the study, as detailed in the "Statistical Analysis" section below.

### Statistical analysis

The data are presented as means and standard deviations (SD), and the differences between *T*1 and *T*2 are presented as means with corresponding 95% confidence intervals [CI]. The proportion of values above the detection range (DR) in ELISA tests is also reported.

A non-inferiority analysis was performed for the primary efficacy outcome: the difference in ΔPpl_max_ between SCT and DEX groups (with a one-sided 97.5% CI obtained by inverting the *t*-test for two-samples assuming equal variances). The normality assumption was verified with Shapiro–Wilk test of normality; the assumption of equal variances was evaluated with the Bartlett test for homogeneity of variances. The sample size calculation was based on the assumption that ΔPpl_max_ was similar in both treatment groups. To obtain a statistical power of 80% with a two-sided *α* = 0.025, ten patients per group were required to determine the non-inferiority of SCT compared to DEX, with a margin of non-inferiority of ≤ 7 cmH_2_O assuming that the SD of differences (end of treatment—baseline) was five in both groups. The non-inferiority margin was determined based on the results reported in Picandet et al. (2003) [49].

The differences between the two treatments were estimated using linear mixed effect models (LME), including random intercept by the horse's ID to account for multiple measurements within the same individual or Tobit (censored) random effect regression models (TRM) for the outcomes with values below DR. Randomization was taken into account by including time and treatment interaction in the model, assuming that the two groups were not different at baseline. Where necessary, the data were log-transformed (using natural logarithms) to meet the assumption of normality. LMEs and TRMs were fitted using the R packages nlme [50] and censReg [51], respectively. All p-values were adjusted for multiple comparisons using the Benjamini and Hochberg method [52] to control the false discovery rate.

Event history analysis was carried out by setting the study's end date at 26th October, 2020. The interval between the end of the treatment protocol, and either the end of the study or the date when clinical signs of asthma reappeared, was calculated for each horse, with the number of days until the end of the study treated as right censored observations. The Kaplan–Meier method was used to estimate the proportion of horses that did not experience a new asthma episode. A log-rank test was used to compare the two treatments.

All analyses were performed with R [53]. The survival analysis was performed with CRAN—Package survival [54]. Adjusted p-values of less than 0.05 were considered statistically significant.

## Results

All horses included in the study completed the study protocol. The SCT group included four mares and six geldings (three Quarter horses, two Lipizzaners, two Icelandic horses, three mix-breeds) with a mean age of 15.5 (3.9) years. The DEX group included seven mares and three geldings (one Quarter horse, two Shetland ponies, one Italian trotter, one Argentine horse, and five mix-breeds) with a mean age of 14.9 (2.9) years.

All horses developed pathognomonic clinical signs after the initial antigen challenge (increased respiratory rate, severe cough, flared nostrils, serous nasal discharge, marked and forced exhalation), which persisted until the start of treatment. Auscultation of the trachea and chest revealed crackles and wheezing. Bacterial or fungal infections were ruled out by BAL cytological evaluation. Reversibility of airway obstruction was confirmed in all horses. No adverse effects were observed in the SCT group during the treatment period. In the DEX group, slight pulsation of the digital arteries and/or warmer hooves on palpation were noted in seven horses during the first few days after starting treatment; however, none of the horses showed discomfort or lameness associated with DEX. The increased pulsation subsided spontaneously after a few days.

### Clinical variables and the cytology of BALf

Non-inferiority of SCT in terms of Δ*P*pl_max_ was not established: ΔPpl_max_ decreased by 9.3 (5.8) cmH_2_O in DEX and by 1.6 (5.4) cmH_2_O in SCT groups (*P* = 0.9, upper bound of one-sided 97.5% CI 13.1).

The clinical score decreased by a value of 3.4 [− 5.464; − 1.303] (*P*_adj_ = 0.01) in the SCT group. The score decreased by an additional 3.1 [− 5.889; − 0.277] points in the DEX group, which, after adjusting for multiple comparisons, was not significantly different from that of SCT (*P*_adj_ = 0.1). The endoscopic score remained unchanged over time in SCT while it decreased in DEX, but this was not significantly different from SCT (Table 1).

**Table 1:** Clinical score and endoscopic score

	SCT mean (SD)		DEX mean (SD)		SCT T2 versus T1 difference		DEX versus SCT for *T*2 and *T*1 difference	
	*T*1	*T*2	*T*1	*T*2	Effect^a^ [95% CI]	*P* value (adjusted)	Effect^b^ [95% CI]	*P* value (adjusted)
CS	17.900 (2.885)	14.450 (3.912)	17.500 (3.197)	11.100 (5.753)	− 3.384 [− 5.464; − 1.303]	0.003 (0.01)	− 3.083 [− 5.889; − 0.277]	0.03 (0.1)
ES*	6.800 (2.418)	6.450 (1.571)	8.400 (1.955)	4.600 (1.243)	0.880 [0.707; 1.096]	0.24 (0.4)	0.670 [0.513; 0.875]	0.005 (0.1)

*CS* clinical score; *ES* endoscopic score; *SCT* Stem cell treatment; *DEX* Dexamethasone treatment; *T1* Beginning of treatment; *T2* End of treatment

^*^Logarithmic transformation (descriptive values are reported on the original scale)

^a^*YT*2–*YT*1 (non-transformed outcome) or *YT*2/*YT*1 (logarithmic transformation) for SCT as estimated with linear mixed effects model

^b^(*YT*2–*YT*1)DEX-(*YT*2–*YT*1)SCT (non-transformed outcome) or (*YT*2/*YT*1)DEX/(*YT*2/*YT*1)SCT (logarithmic transformation) as estimated with linear mixed effects model. Additional data are available in graphical form in the Additional file 1: Figure S1

The mean TCC of the BALf did not differ significantly over time in SCT. Also, the mean TCC did not differ between treatments. Similar results were obtained in the differential leukocyte counts (Table 2).

**Table 2:** Bronchoalveolar lavage fluid cytology

	SCT mean (SD)		DEX mean (SD)		SCT T2 versus T1 difference		DEX versus SCT for T2 and T1 difference	
	*T*1	*T*2	*T*1	*T*2	Effect^a^ [95% CI]	P value (adjusted)	Effect^b^ [95% CI]	*P* value (adjusted)
TCC*	0.437 (0.256)	0.483 (0.261)	0.650 (0.382)	0.540 (0.359)	1.033 [0.681; 1.568]	0.87 (0.9)	0.848 [0.492; 1.463]	0.53 (0.7)
Mac (%)	34.580 (4.693)	34.510 (7.563)	27.930 (7.932)	35.520 (10.653)	2.665 [-3.611; 8.941]	0.38 (0.5)	2.190 [-5.452; 9.831]	0.60 (0.7)
Neut (%)*	33.950 (11.060)	31.310 (14.240)	39.550 (24.475)	33.820 (16.305)	0.886 [0.674; 1.164]	0.36 (0.5)	1.058 [0.733; 1.526]	0.75 (0.8)
Lymph (%)	31.390 (9.217)	31.550 (10.630)	29.460 (17.911)	32.860 (14.146)	0.527 [-6.740; 7.795]	0.88 (0.9)	2.506 [-7.217; 12.228]	0.59 (0.7)
Eos (%)	0.870 (1.421)	1.930 (3.804)	0.580 (1.573)	0.000 (0.000)	1.148 [-0.299; 2.595]	0.11 (0.3)	-1.816 [-3.670; 0.038]	0.05 (0.2)
Mast (%)	0.410 (0.534)	0.910 (1.249)	0.620 (1.219)	0.180 (0.569)	0.406 [-0.337; 1.148]	0.27 (0.4)	-0.751 [-1.635; 0.133]	0.09 (0.2)

TCC (WBC/mm^3^), mean total cell count (BALf)

*Mac* macrophages, *Neut* neutrophils, *Lymph* lymphocytes, *Eos* eosinophils, *Mast* mast cells, *SCT* Stem cell treatment, *DEX* Dexamethasone treatment, *T1* Beginning of treatment, *T2* End of treatment

*Logarithmic transformation (descriptive values are reported on the original scale)

^a^*YT*2–*YT*1 (non-transformed outcome) or *YT*2/*YT*1 (logarithmic transformation) for SCT as estimated with linear mixed effects model

^b^(*YT*2–*YT*1)DEX-(*YT*2–*YT*1)SCT (non-transformed outcome) or (*YT*2/*YT*1)DEX/(*YT*2/*YT*1)SCT (logarithmic transformation) as estimated with linear mixed effects model. Additional data are available in graphical form in the Additional file 2: Figure S2

### Relative quantification of the mRNA and protein concentration of cytokines

The mRNA quantification was successful for all cytokines, whereas the detection of cytokine proteins (by ELISA) was successful only for IL-1β, IL-4 and TNFα. Other cytokine concentrations were below the DR of the respective ELISA tests. The mRNA ratio of most cytokines remained stable over time in SCT and DEX while that of IL-17 decreased over time in SCT (*P*_adj_ = 0.05). The mRNA ratio of IL-17 also decreased in DEX, which was not significantly different from SCT (*P*_adj_ = 0.2), (Table 3).

**Table 3:** Relative quantification of cytokines' mRNA and their protein concentration

	SCT mean (SD) [% > DR]		DEX mean (SD) [% > DR]		SCT T2 versus T1 difference		DEX versus SCT for T2 and T1 difference	
	*T*1	*T*2	*T*1	*T*2	Effect^a^ [95% CI]	P value (adjusted)	Effect^b^ [95% CI]	P value (adjusted)
IL-1β*	22.510 (17.260)	24.078 (34.070)	27.651 (30.293)	11.089 (16.941)	0.608 [0.281; 1.315]	0.19 (0.4)	0.472 [0.171; 1.307]	0.14 (0.3)
IL-4*	0.140 (0.106)	0.190 (0.385)	0.116 (0.122)	0.182 (0.359)	0.812 [0.329; 2.003]	0.6 (0.7)	0.796 [0.252; 2.510]	0.7 (0.8)
IL-8*	7470.490 (5017.252)	8013.460 (11,453.562)	10,222.300 (13,818.670)	5742.500 (4475.492)	0.607 [0.296; 1.245]	0.2 (0.4)	1.137 [0.445; 2.905]	0.8 (0.8)
IL-17*	0.019 (0.014)	0.009 (0.010)	0.020 (0.021)	0.005 (0.008)	0.409 [0.201; 0.831]	**0.02 (0.05)**	0.432 [0.167; 1.120]	0.08 (0.2)
TNFα*	146.908 (140.452)	112.709 (119.865)	113.907 (67.441)	78.013 (48.822)	0.780 [0.487; 1.250]	0.3 (0.4)	0.803 [0.442; 1.458]	0.4 (0.7)
INFy*	2.831 (3.534)	1.610 (1.330)	2.113 (2.654)	1.551 (1.587)	0.665 [0.342; 1.290]	0.2 (0.4)	0.782 [0.330; 1.850]	0.5 (0.7)
eIL-1β*	370.062 (293.004) [90]	279.188 (156.496) [90]	217.321 (84.624) [80]	203.012 (84.247) [60]	0.699 [0.586; 0.833]	** < 0.001 (< 0.001)**	0.828 [0.678; 1.011]	0.06 (0.2)
eIL-4*	340.817 (291.316) [100]	257.304 (165.533) [100]	198.584 (97.086) [90]	203.012 (84.247) [60]	0.555 [0.422; 0.731]	** < 0.001 (< 0.001**)	0.676 [0.482; 0.949]	**0.02 (0.1)**
eTNFα*	1102.100 (878.340) [100]	740.418 (759.764) [100]	609.070 (606.676) [100]	473.075 (378.766) [100]	0.553 [0.376; 0.812]	**0.005 (0.02)**	1.765 [1.033; 3.014]	**0.04 (0.1)**

Significant values are in bold

*e* ELISA determined concentration of cytokine, *IL* Interleukine, *SCT* Stem cell treatment, *DEX* Dexamethasone treatment, *DR* Detection range for ELISA assays, % > *DR* proportion of variables above DR, *T1* Beginning of treatment, *T2* End of treatment

^*^Logarithmic transformation (descriptive values are on the original scale)

^a^YT2-YT1 (non-transformed outcome) or YT2/YT1 (logarithmic transformation) for SCT as estimated with linear mixed effects model or Tobit regression

^b^(YT2-YT1)DEX-(YT2-YT1)SCT (non-transformed outcome) or (YT2/YT1)DEX/(YT2/YT1)SCT (logarithmic transformation) as estimated with linear mixed effects model or Tobit random effects model. Additional data are available in graphical form in the Additional file 3: Figure S3

The concentration of the cytokines IL-1β (*P*_adj_ = 0.001), IL-4 (*P*_adj_ = 0.001) and TNFα (*P*_adj_ = 0.02) decreased significantly in SCT. A decrease in IL-1β, IL-4 and TNFα concentrations was also observed in DEX, which did not differ significantly from SCT (Table 3).

### Long-term outcome

**Fig. 2:**
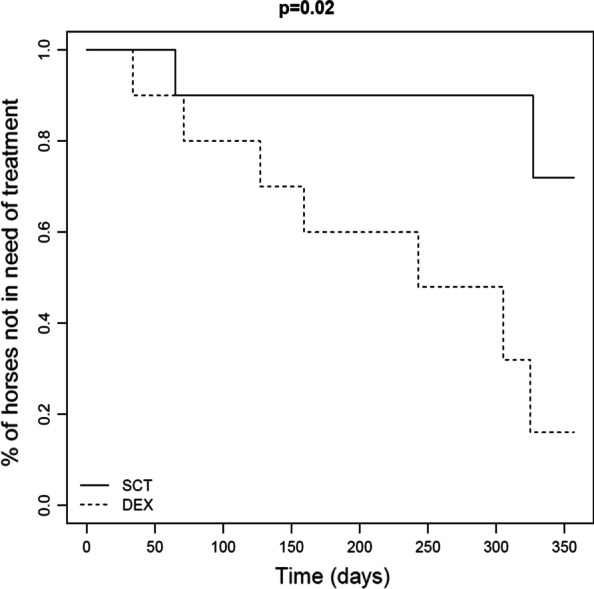
Long-term outcome. The timepoint expressed in number of days after completion of treatment when horses re-developed signs of SEA and required treatment. SCT, Stem cell treatment; DEX, Dexamethasone treatment; *p* value

In the follow-up phase it took longer until horses treated with MSC had an exacerbation of SEA. Exacerbations of SEA were documented in the DEX group shortly after discontinuation of treatment, while most horses in the SCT group remained stable for at least 300 days after discontinuation of SCT (*P* = 0.02) (Fig. 2).

## Discussion

In this study, the intrabronchial application of autologous AD-MSC had a modest short-term therapeutic effect and a possible positive long-term effect on SEA. Short-term effects were expressed in a reduction of the CS score, a reduced expression of IL-17 and reduced concentrations of IL-1β, IL-4 and TNFα in BALf. Several other variables showed a tendency to improve in the SCT group, which was similar to DEX treatment. The long-term effect of MSC observed in our study is consistent with the results of Trzil et al. (2016), who reported reduced AHR and airway remodeling, and a reduction in inflammation nine months after starting therapy in cats [31]. Treatment with BMMC may also have a positive immunomodulatory effect in SEA, as reported in a small group of horses [34].

In our study, AD-MSC were used. Adipose tissue- and bone marrow-derived MSC have a similar surface immunophenotype and differentiation potential [55–57] and appear to be similarly effective on fibrosis-promoting pathways [30]. The anti-inflammatory effect of MSC is achieved through the regulation of Th1 and Th2 cytokines [23], mainly through their induction of CD4 regulatory T cells (Treg) [24–26]. MSC also manipulate their environment through paracrine actions, which directly promotes cell and tissue repair [24, 58, 59]. Adipose-derived MSCs were classified based on adhesion to plastic, morphology, and trilineage differentiation [60]. Equine AD-MSC, cultured in basal growth medium, adhered to the bottom of plastic culture dishes and showed typical fibroblast-like morphology. Since no specific data or guidelines for the determination of surface markers of equine MSCs have been published to date, this was not pursued in this study.

Intrabronchial administration of biological drugs allows direct access to and more rapid interaction with the immune system of the lungs. Compared to systemic therapy, fewer cells are required and the possibility of potentially harmful systemic side effects of treatment is lower [21]. Therefore, AD-MSC were administered intrabronchially in this study. To investigate the effect of MSC therapy on SEA-related pulmonary inflammation, MSC treatment was compared with dexamethasone treatment. Constant antigenic challenge promotes neutrophil accumulation in the airways of SEA affected horses [1, 2], leading to upregulation of several inflammatory pathways and the production of pro-inflammatory cytokines [61–67]. The pathogenic process of SEA operates via positive feedback loops that promote persistent inflammation [62]. Dexamethasone has an immunosuppressive effect that inhibits the activation of the positive feedback loops [68–70]. Therefore, DEX serves as an appropriate control to study the effect of SCT.

The expression of pro-inflammatory cytokines (IL-1β, IL-8 and TNFα) and cytokine representatives of the immune responses of type 1 (Th1: IFNγ), type 2 (Th2: IL -4) and type 17 (Th17: IL-17) were investigated to assess different pathways of the immune response. The study design required that the horses remain in an exacerbated SEA state while AD-MSC were cultured. Therefore, cytokine expression in this study was the reflection of a steady-state SEA. This is most likely the reason why at the time of sampling the cytokines IL-4, IL-17 and IFNγ, which are crucial especially in the initial phase of inflammation [65, 66, 71], were somehow lower compared to other cytokines.

Stem cell treatments in other studies also showed variable results in terms of cytokine gene expression and translation, but consistently demonstrated a reduction in airway inflammation and parenchymal damage [22–24]. Cho et al. (2014) reported a reduction of inflammation and symptoms in the mouse asthma model following intravenous administration of AD-MSC. The treatment inhibited the expression of Th2 cytokines and increased the expression of Th1 cytokines in BALf [27]. Similar results were reported by Mariñas-Pardo et al. (2014) after intravenous administration of labeled AD-MSC rapidly downregulated airway inflammation associated with Th1 cytokines. It is important to note that MSC treatment effects decreased under sustained allergen challenge, although MSC were still present in the lungs 72 h and two weeks after administration [28]. Other similar studies have shown that MSC are short-lived and cannot be detected 24 h after infusion [72, 73], although even after phagocytosed by macrophages, they are thought to continue to modulate the adaptive immune response [73]. Thus, the therapeutic effect of MSC is most likely not related to their viability in local tissues. Intrabronchial delivery of biologic therapeutics is also considered advantageous over systemic delivery, as fewer cells are required and potential adverse systemic side effects of treatment are reduced [21]. It also allows direct access to and faster interaction with the local pulmonary immune system. These were the reasons for choosing intrabronchial MSC administration in this study.

The pathophysiology of inflammatory processes in SEA is not fully understood. An interaction of Th1, Th2 and Th17 differentiation pathways has been proposed to play a crucial role in a chronic asthma model [1, 2, 74–76]. In this study, IL-1β, TNFα and IL-8 mRNA was readily detected in all horses at T1. Treatment with MSC influenced the expression of IL-17, a pro-inflammatory cytokine that plays a crucial role in triggering inflammatory responses, including autoimmune diseases. The exact role and underlying mechanism of dysregulated production of IL-17 and the inflammation triggered by IL-17 are still not well understood [77]. It is important to note that TNFα and IL-1β are, among some other cytokines, the downstream target genes of IL-17 [78], which may also be the reason for a decrease in TNFα and IL-1β concentrations in BALf. We could not pinpoint the reason for the attenuation of the transcription of IL-17 mRNA in this study. It was reported that microRNA (miRNA) expression strongly influences the concentrations of IL-17 [79]. Stem cell treatment may cause differential expression of miRNA, which may affect the transcription of IL-17 mRNA [79, 80]. IL-17 is most likely a critical cytokine that combines T cell activation with the mobilization of neutrophils [81] and indirectly promotes their chemotaxis and activation [35]. The decreased expression of IL-17 may have significantly contributed to the improvement of CS and the long-term positive effect of MSC treatment. The effect of dexamethasone treatment on IL-17 expression was even more evident; however, the immunomodulatory pathways of both treatments are different [82].

It was not possible to define how MSC treatment affected mRNA stability and translation as well as the post-transcriptional stability of cytokines. The mRNA translation was only detected for IL-1β, IL-4 and TNFα, all of which were significantly decreased in BALf in SCT. IL-1β synthesis is controlled both at the transcriptional and post-transcriptional or translational levels [83, 84]. Based on our results, IL-1β levels were most likely reflective of reduced mRNA translation or post-translational attenuation of the protein, since the IL-1β mRNA remained stable from T1 to T2. The same would also apply to IL-4. Relatively low values of IL-4 mRNA and high concentrations of IL-4 in BALf do not agree with the data of other researchers who reported a good correlation between the mRNA and the amount of IL-4 protein secretion in biological samples [85]. The presence of IL-4 in human samples indicates a predominance of a Th2 immune response [86, 87]. The Th2 immune response further modulates the mRNA stability in a positive feedback loop [85, 88, 89]. This could explain the low stability of IL-4 mRNA in the asthmatic lung of horses since the pathogenesis of the disease is different and Th2 polarization is not predominant in horses. Low levels of IL-4 mRNA can still produce high levels of protein because multiple molecules of protein are translated from each mRNA molecule [90].

TNFα mRNA [91–93] and protein [91, 92] have short half-lives and low stability in systemic circulation and biological samples. Therefore the high levels of both in our samples were surprising. These results differ from the results of Montgomery et al. [2018), who found low values of the TNFα protein in the respiratory tract of asthmatic horses [94]. The regulatory mechanisms controlling the synthesis of TNFα at the post-transcriptional level depend on cell types and cell activation pathways [95, 96]. In general, macrophage activation stimulates the translation of TNFα, which is otherwise blocked, resulting in high TNFα levels [96]. It appears that MSC treatment may affect the post-transcriptional and translational phases of TNFα gene expression.

The mRNA expression was not always associated with detectable concentrations of cytokine proteins (by ELISA) in this study. Differences in detection can be attributed to the variable stability of mRNAs. This is a known characteristic when biological samples are analyzed by both methods [97–99]. Most studies report a strong association between IFNγ mRNAs and the protein concentration and a more variable association between TNFα and IL-8 mRNAs and their proteins. Expression of mRNA and protein concentration also have limited correlation for IL-1β, IL-4 and IL-17 [98]. Other regulatory mechanisms may influence cytokine gene expression, some of which are related to post-transcriptional regulation and the silencing of mRNAs by miRNAs, as discussed above [79].

The undetectable values (value below the ELISA detection range) of the IL-8 protein in BALf were surprising. IL-8 gene expression is often markedly increased in BALf samples from asthmatic horses [66, 100, 101]. The reasons for this may be attributed to the post-transcriptional, pre-, and post-translational control of the transcript. IL-8 mRNA is very stable in biological samples [93, 102]; however, it has been reported that the IL-8 protein has relatively short stability in plasma and probably also in BALf [103]. In addition, IL-8 receptors are densely expressed on neutrophils [104, 105], which are extremely prevalent in the inflammatory environment of the asthmatic lungs of horses. The IL-8 protein probably binds neutrophils shortly after mRNA translation and may therefore no longer be detectably by ELISA [104]. It is also possible that the IL-8 translation was attenuated by miRNA-17 [106], which may be abundant in asthma [107].

The long-term effect of treatments was determined based on the redevelopment of pathognomonic clinical signs for SEA. The use of diagnostic modalities used in T1 and T2 for long-term follow-up would likely provide some additional information. However, such a study model would require a population-based approach with a more representative number of participants to account for non-standardized investigation time-points, husbandry conditions and animal use protocols.

In this study, we did not evaluate the lung tissue after treatment; therefore, any structural changes and remodeling of lung tissue could not be determined. Repeated lung biopsies were not within the scope of this study and would likely trigger inflammatory pathways unrelated to SEA.

## Conclusions

This study has identified several positive effects of MSC treatment in SEA. Some changes were captured by assessment after a short-term, but most pronounced was the long-term effect on SEA, which was strikingly different for horses in the SCT compared to the DEX group. It is important to note, however, that SCT did not preclude recurrence of the clinical signs of SEA in all horses at one year. Like several other studies concerning regenerative therapy, we could not define the exact effects of MSC treatment on the development and persistence of asthma. The results of this study suggest that positive effects of treatment with MSC on SEA are possible and that the horse may be a suitable model to thoroughly investigate the effects of MSC treatment in asthma and possibly other chronic diseases.

## Supplementary Information

**Additional file 1**: Figure S1. Clinical and endoscopic scores. CS, clinical score; ES, endoscopic score; SCT, Stem cell treatment; DEX, Dexamethasone treatment; T1, Beginning of treatment; T2, End of treatment; log, logarithmic transformation.

**Additional file 2**: Figure S2. Bronchoalveolar lavage fluid cytology (x109/L). TCC, mean total cell count; Mac, macrophages; Neut, neutrophils; Lymph, lymphocytes; Eos, eosinophils; Mast, mast cells; SCT, Stem cell treatment; DEX, Dexamethasone treatment; T1, Beginning of treatment; T2, End of treatment; log, logarithmic transformation

**Additional file 3**: Figure S3. Relative quantification of cytokines' mRNA and their protein concentration. e, ELISA determined concentration of cytokine; IL, Interleukin; SCT, Stem cell treatment; DEX, Dexamethasone treatment; T1, Beginning of treatment; T2, End of treatment; log, logarithmic transformation

**Additional file 4**: List of primer sequences used for qRT-PCR

## Data Availability

The datasets used and/or analysed during the current study are available from the corresponding author on reasonable request.

## Abbreviations

AD-MSC: Adipose-derived mesenchymal stem cells
AHR: Airway hyperreactivity
BAL: Bronchoalveolar lavage
BALf: Bronchoalveolar lavage fluid
BMMC: Bone marrow-derived mononuclear cells
BW: Body weight
CD4: Cluster of differentiation 4
CI: Confidence interval
CS: Clinical score
cDNA: Complementary deoxyribonucleic acid
DEX: Dexamethasone treatment group
DMEM: Dulbecco – modified eagle medium
DR: Detection range
ELISA: Enzyme-linked immunosorbent assay
ES: Endoscopic score
FBS: Fetal bovine serum
GAPDH: Glyceraldheyde 3-phosphate dehydrogenase
IFNγ: Interferon gamma
IL: Interleukine
LME: Linear mixed effects
MSC: Mesenchymal stem cells
PBS: Phosphate buffered saline
qPCR: Quantitative polymerase chain reaction
ΔPpl_max_: Maximum change in pleural pressure during the respiratory cycle
PO: Per os treatment
RNA: Ribonucleic acid
mRNA: Messenger RNA
miRNA: Micro-RNA
RT-qPCR: Reverse transcription-quantitative polymerase chain reaction
SD: Standard deviation
SEA: Severe equine asthma
SCT: Stem cell treatment group
TCC: Total cell count
Th: T-helper cells
TNFα: Tumor necrosis factor alpha
Treg: Regulatory T cells
TRM: Tobit random effect regression model
T1: Time 1, beginning of treatment
T2: Time 2, end of treatment
