# Supplementary figures and images for: Effect of intrabronchial administration of autologous adipose-derived mesenchymal stem cells on severe equine asthma
Source: Stem Cell Res Ther. 2022 Jan 21;13:23. doi: 10.1186/s13287-022-02704-7 (PMC8777441; doi:10.1186/s13287-022-02704-7)

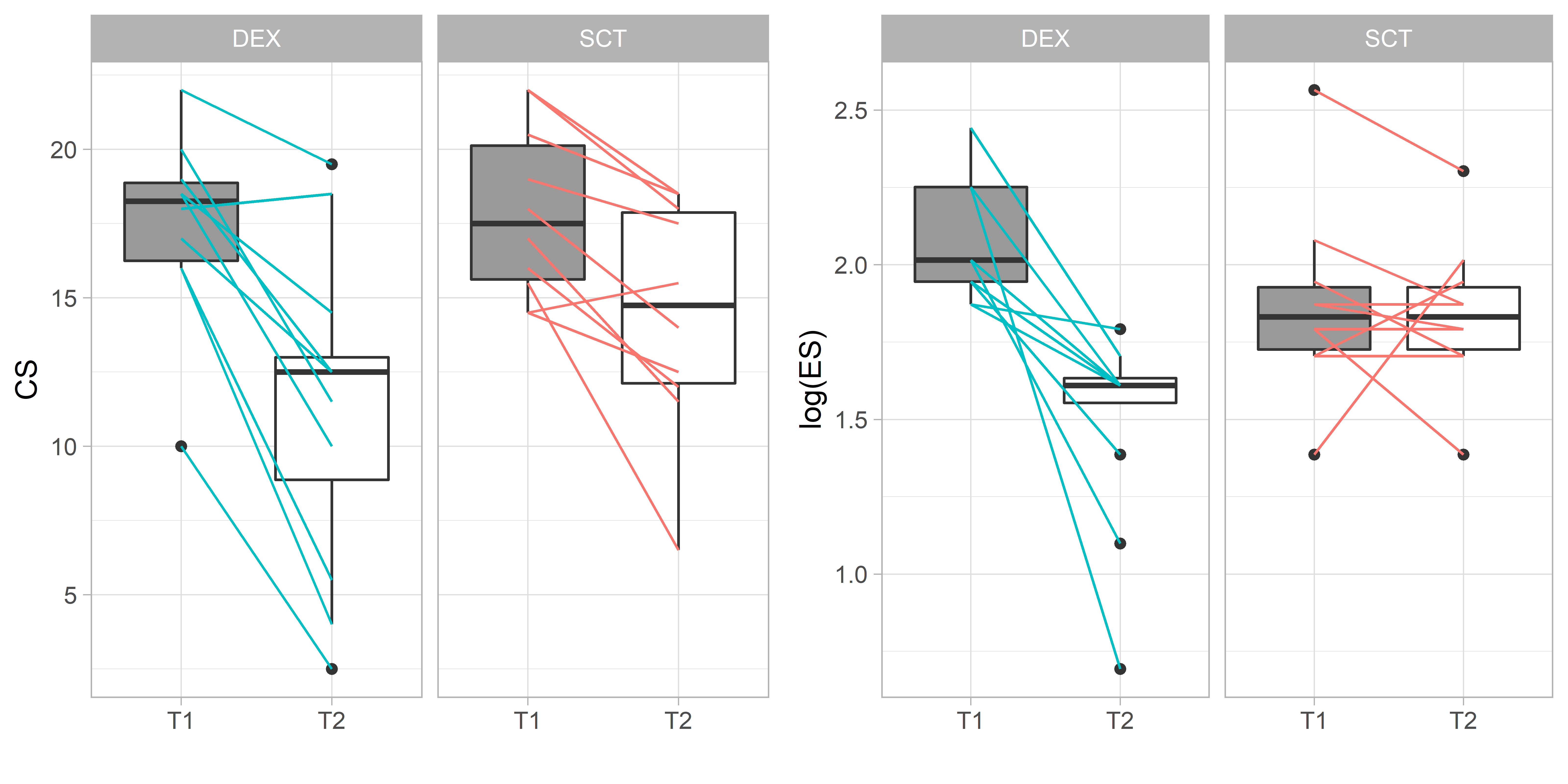

Supplement: Supplementary file 1 — Additional file 1: Figure S1. Clinical and endoscopic scores. CS, clinical score; ES, endoscopic score; SCT, Stem cell treatment; DEX, Dexamethasone treatment; T1, Beginning of treatment; T2, End of treatment; log, logarithmic transformation. [file 13287_2022_2704_MOESM1_ESM.tif]

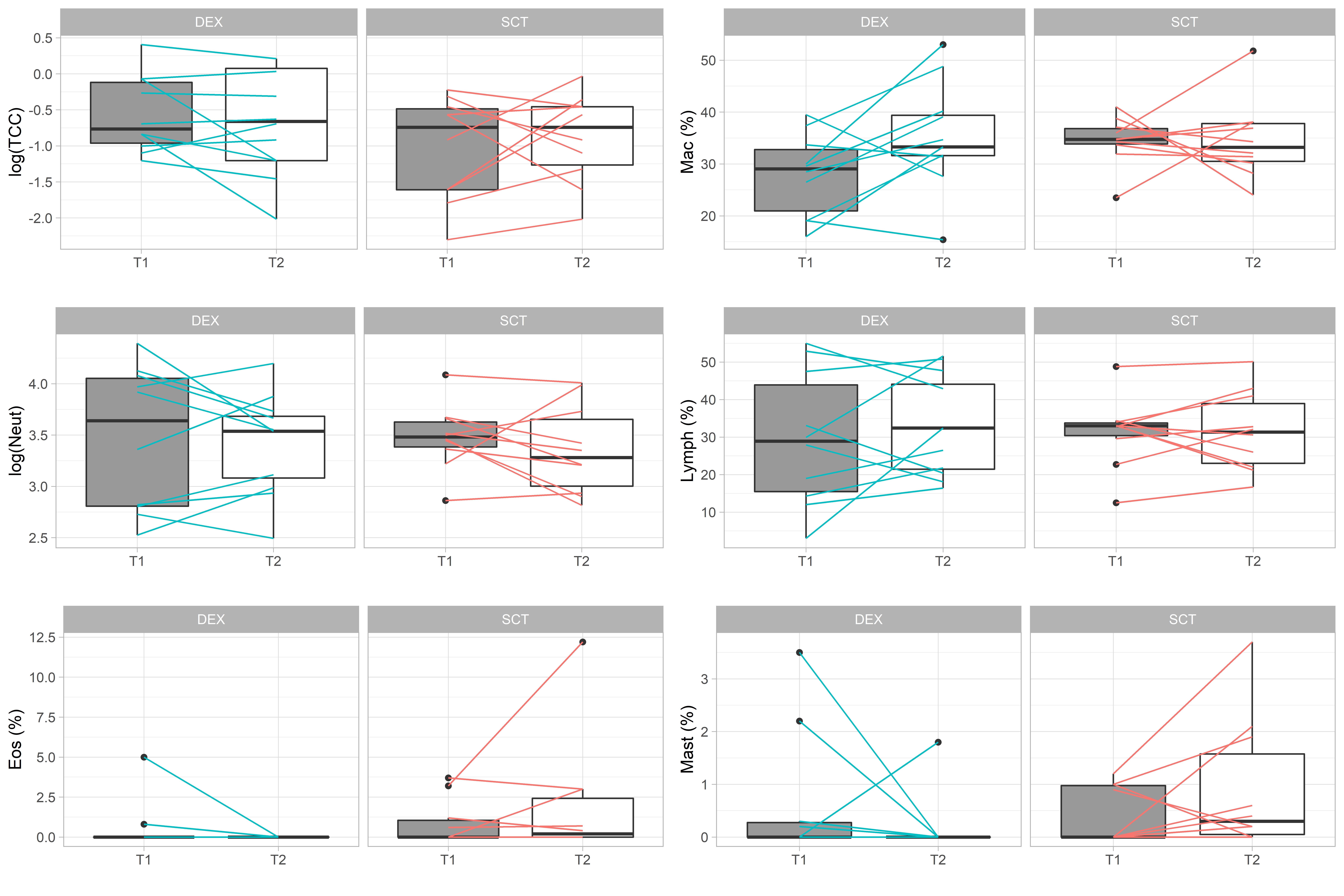

Supplement: Supplementary file 2 — Additional file 2: Figure S2. Bronchoalveolar lavage fluid cytology (x109/L). TCC, mean total cell count; Mac, macrophages; Neut, neutrophils; Lymph, lymphocytes; Eos, eosinophils; Mast, mast cells; SCT, Stem cell treatment; DEX, Dexamethasone treatment; T1, Beginning of treatment; T2, End of treatment; log, logarithmic transformation [file 13287_2022_2704_MOESM2_ESM.tif]

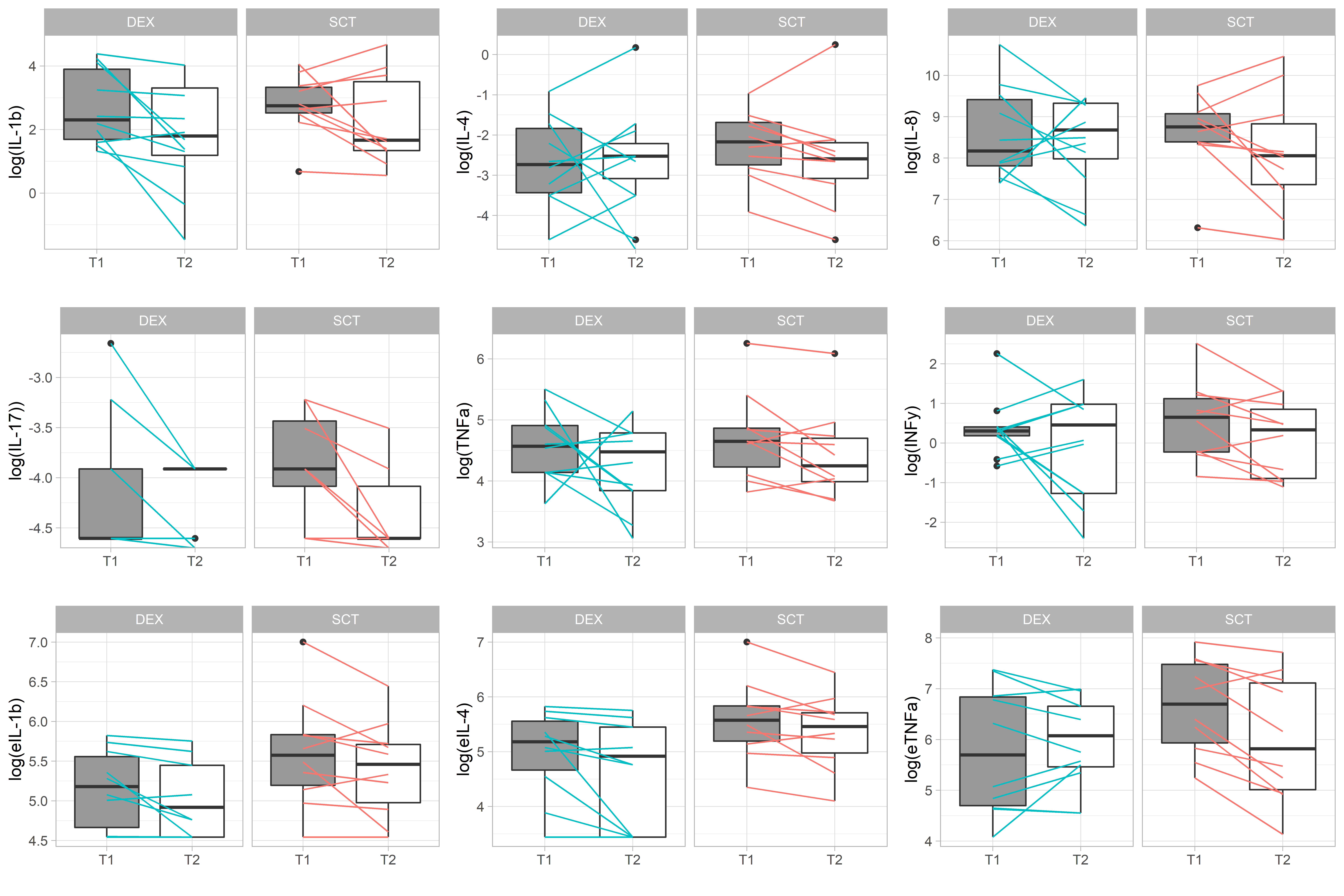

Supplement: Supplementary file 3 — Additional file 3: Figure S3. Relative quantification of cytokines' mRNA and their protein concentration. e, ELISA determined concentration of cytokine; IL, Interleukin; SCT, Stem cell treatment; DEX, Dexamethasone treatment; T1, Beginning of treatment; T2, End of treatment; log, logarithmic transformation [file 13287_2022_2704_MOESM3_ESM.tif]
