# Supplementary material for: Effect of intrabronchial administration of autologous adipose-derived mesenchymal stem cells on severe equine asthma
Source: Stem Cell Res Ther. 2022 Jan 21;13:23. doi: 10.1186/s13287-022-02704-7 (PMC8777441; doi:10.1186/s13287-022-02704-7)
Supplement: Supplementary file 4 — Additional file 4: List of primer sequences used for qRT-PCR [file 13287_2022_2704_MOESM4_ESM.docx]

| Gene | Context Sequence | Assay ID | NCBI Gene Reference |
| --- | --- | --- | --- |
| **IL-1β** | TTCAAGCATGTCGTGTCAATCATTG | Ec04260298_s1 | NM_001082526.1 |
| **IL-4** | GCATGGCAAACGGGACCTGCTGTAC | Ec03468790_m1 | NM_001082519.1 |
| **IL-8** | TTGAAGAGAGCTGAGGGGCAAAATC | Ec03468860_m1 | NM_001083951.1 |
| **IL-17** | TTGGAATCTCCACCGCAACGAGGAC | Ec03470096_m1 | NM_001143792.1 |
| **TNFα** | GCCCATGTTGTAGCAAACCCCCAAG | Ec03467871_m1 | NM_001081819.1 |
| **IFNγ** | CTGATTCAGATTCCGGTAAATGATC | Ec03468606_m1 | NM_001081949.1 |
| **GAPDH** | CCTGAAGATTGTCAGCAATGCCTCC | Ec03210916_gH | NM_001163856.1 |

List of primer sequences used for qRT-PCR (Thermo Fisher Scientific, USA, cat.no: [4331182](https://www.thermofisher.com/order/catalog/product/4331182)).
